# Supplementary material for: Pyruvate Dehydrogenase Kinase 1 inhibition mediated oxidative phosphorylation enhancement in cartilage promotes osteoarthritis progression
Source: BMC Musculoskelet Disord. 2023 Jul 20;24:597. doi: 10.1186/s12891-023-06585-6 (PMC10357736; doi:10.1186/s12891-023-06585-6)
Supplement: Supplementary file 1 — Additional file 1. [file 12891_2023_6585_MOESM1_ESM.zip › KEGG.Gsea.1653622432464/KEGG_HOMOLOGOUS_RECOMBINATION.html]

Details for gene set KEGG\_HOMOLOGOUS\_RECOMBINATION[GSEA]

|  || Dataset | 55457\_collapsed\_to\_symbols.cls.cls #OA\_versus\_normal.cls.cls #OA\_versus\_normal\_repos |
| Phenotype | cls.cls#OA\_versus\_normal\_repos |
| Upregulated in class | OA |
| GeneSet | KEGG\_HOMOLOGOUS\_RECOMBINATION |
| Enrichment Score (ES) | 0.33245188 |
| Normalized Enrichment Score (NES) | 1.1554765 |
| Nominal p-value | 0.25467288 |
| FDR q-value | 0.40683743 |
| FWER p-Value | 1.0 |
Table: GSEA Results Summary

  

Fig 1: Enrichment plot: KEGG\_HOMOLOGOUS\_RECOMBINATION      
 Profile of the Running ES Score & Positions of GeneSet Members on the Rank Ordered List

  

| SYMBOL | TITLE | RANK IN GENE LIST | RANK METRIC SCORE | RUNNING ES | CORE ENRICHMENT || 1 | RPA3 | replication protein A3 [Source:HGNC Symbol;Acc:HGNC:10291] | 13 | 1.159 | 0.0959 | Yes |
| 2 | RPA2 | replication protein A2 [Source:HGNC Symbol;Acc:HGNC:10290] | 339 | 0.727 | 0.1315 | Yes |
| 3 | RPA1 | replication protein A1 [Source:HGNC Symbol;Acc:HGNC:10289] | 571 | 0.632 | 0.1664 | Yes |
| 4 | POLD1 | "DNA polymerase delta 1, catalytic subunit [Source:HGNC Symbol;Acc:HGNC:9175]" | 811 | 0.566 | 0.1952 | Yes |
| 5 | POLD4 | "DNA polymerase delta 4, accessory subunit [Source:HGNC Symbol;Acc:HGNC:14106]" | 820 | 0.564 | 0.2417 | Yes |
| 6 | RAD51B | RAD51 paralog B [Source:HGNC Symbol;Acc:HGNC:9822] | 893 | 0.545 | 0.2817 | Yes |
| 7 | RAD51C | RAD51 paralog C [Source:HGNC Symbol;Acc:HGNC:9820] | 1027 | 0.512 | 0.3142 | Yes |
| 8 | RAD51 | RAD51 recombinase [Source:HGNC Symbol;Acc:HGNC:9817] | 1581 | 0.416 | 0.3060 | Yes |
| 9 | XRCC3 | X-ray repair cross complementing 3 [Source:HGNC Symbol;Acc:HGNC:12830] | 1902 | 0.372 | 0.3123 | Yes |
| 10 | SEM1 | SEM1 26S proteasome subunit [Source:HGNC Symbol;Acc:HGNC:10845] | 2029 | 0.358 | 0.3325 | Yes |
| 11 | SSBP1 | single stranded DNA binding protein 1 [Source:HGNC Symbol;Acc:HGNC:11317] | 3038 | 0.246 | 0.2747 | No |
| 12 | BRCA2 | BRCA2 DNA repair associated [Source:HGNC Symbol;Acc:HGNC:1101] | 3853 | 0.170 | 0.2257 | No |
| 13 | NBN | nibrin [Source:HGNC Symbol;Acc:HGNC:7652] | 4136 | 0.143 | 0.2158 | No |
| 14 | MUS81 | MUS81 structure-specific endonuclease subunit [Source:HGNC Symbol;Acc:HGNC:29814] | 4139 | 0.143 | 0.2276 | No |
| 15 | BLM | BLM RecQ like helicase [Source:HGNC Symbol;Acc:HGNC:1058] | 4843 | 0.084 | 0.1800 | No |
| 16 | RAD54B | RAD54 homolog B [Source:HGNC Symbol;Acc:HGNC:17228] | 5753 | 0.012 | 0.1103 | No |
| 17 | POLD2 | "DNA polymerase delta 2, accessory subunit [Source:HGNC Symbol;Acc:HGNC:9176]" | 6311 | -0.028 | 0.0694 | No |
| 18 | RAD50 | RAD50 double strand break repair protein [Source:HGNC Symbol;Acc:HGNC:9816] | 6474 | -0.040 | 0.0602 | No |
| 19 | RAD51D | RAD51 paralog D [Source:HGNC Symbol;Acc:HGNC:9823] | 6815 | -0.064 | 0.0392 | No |
| 20 | POLD3 | "DNA polymerase delta 3, accessory subunit [Source:HGNC Symbol;Acc:HGNC:20932]" | 7568 | -0.125 | -0.0088 | No |
| 21 | MRE11 | "MRE11 homolog, double strand break repair nuclease [Source:HGNC Symbol;Acc:HGNC:7230]" | 11458 | -0.497 | -0.2694 | No |
| 22 | TOP3B | DNA topoisomerase III beta [Source:HGNC Symbol;Acc:HGNC:11993] | 11555 | -0.508 | -0.2344 | No |
| 23 | RAD52 | "RAD52 homolog, DNA repair protein [Source:HGNC Symbol;Acc:HGNC:9824]" | 12134 | -0.627 | -0.2268 | No |
| 24 | RAD54L | RAD54 like [Source:HGNC Symbol;Acc:HGNC:9826] | 12204 | -0.646 | -0.1781 | No |
| 25 | RPA4 | replication protein A4 [Source:HGNC Symbol;Acc:HGNC:30305] | 12406 | -0.709 | -0.1344 | No |
| 26 | TOP3A | DNA topoisomerase III alpha [Source:HGNC Symbol;Acc:HGNC:11992] | 12707 | -0.867 | -0.0852 | No |
| 27 | XRCC2 | X-ray repair cross complementing 2 [Source:HGNC Symbol;Acc:HGNC:12829] | 12882 | -1.198 | 0.0014 | No |
Table: GSEA details [plain text format]

  

Fig 2: KEGG\_HOMOLOGOUS\_RECOMBINATION      
 Blue-Pink O' Gram in the Space of the Analyzed GeneSet

  

Fig 3: KEGG\_HOMOLOGOUS\_RECOMBINATION: Random ES distribution      
 Gene set null distribution of ES for **KEGG\_HOMOLOGOUS\_RECOMBINATION**
